# Allergic *Aspergillus* Rhinosinusitis

**DOI:** 10.3390/jof2040032

**Published:** 2016-12-08

**Authors:** Arunaloke Chakrabarti, Harsimran Kaur

**Affiliations:** Department of Medical Microbiology, Postgraduate Institute of Medical Education and Research (PGIMER), Chandigarh 160012, India; drharsimranpgi@gmail.com

**Keywords:** fungal sinusitis, allergy, *Aspergillus*, dematiaceous fungi, epidemiology, pathogenesis, diagnosis, management

## Abstract

Allergic fungal rhinosinusitis (AFRS) is a unique variety of chronic polypoid rhinosinusitis usually in atopic individuals, characterized by presence of eosinophilic mucin and fungal hyphae in paranasal sinuses without invasion into surrounding mucosa. It has emerged as an important disease involving a large population across the world with geographic variation in incidence and epidemiology. The disease is surrounded by controversies regarding its definition and etiopathogenesis. A working group on “Fungal Sinusitis” under the International Society for Human and Animal Mycology (ISHAM) addressed some of those issues, but many questions remain unanswered. The descriptions of “eosinophilic fungal rhinosinusitis” (EFRS), “eosinophilic mucin rhinosinusitis” (EMRS) and mucosal invasion by hyphae in few patients have increased the problem to delineate the disease. Various hypotheses exist for etiopathogenesis of AFRS with considerable overlap, though recent extensive studies have made certain in depth understanding. The diagnosis of AFRS is a multi-disciplinary approach including the imaging, histopathology, mycology and immunological investigations. Though there is no uniform management protocol for AFRS, surgical clearing of the sinuses with steroid therapy are commonly practiced. The role of antifungal agents, leukotriene antagonists and immunomodulators is still questionable. The present review covers the controversies, recent advances in pathogenesis, diagnosis, and management of AFRS.

## 1. Introduction

The term rhinosinusitis refers to the inflammation of nasal and paranasal sinus mucosa caused by either infectious (bacterial or fungal) or non-infectious (allergic or non-allergic or immunological) causes [1]. Fungal rhinosinusitis (FRS) is defined as the rhinosinusitis where fungi are responsible for causing the immunopathogenesis. The disease impairs the quality of life and creates socioeconomic loss. Due to several hypotheses surrounding FRS, the understanding of the disease is still evolving, though it is being recognized as an emerging disease entity. The allergic fungal rhinosinusitis is a subset of FRS with complex immune modulation in its pathogenesis. Allergic fungal rhinosinusitis has several challenges due to its controversies in definition and pathogenesis, though extensive studies have been conducted in recent years. The uniform diagnostic and management guidelines of the disease will not be possible until the controversies are resolved. It is therefore important to have a comprehensive review on every aspect of the disease. The present review covers the controversies, recent advances in pathogenesis, diagnosis and management of AFRS to give the readers a comprehensive update on this topic.

## 2. Historical Account

Fungal rhinosinusitis was described for the first time in 1791 by Plaignaud in a 22-year-old male suffering from maxillary pain [2,3]. Thereafter, Schubert in 1885 and Mackenzie in 1894 described cases of a non-invasive form of paranasal rhinosinusitis [4,5]. In 1897, Oppe mentioned the possibility of an invasive variety of *Aspergillus* rhinosinusitis [6]. Baker et al. in 1957 finally described an acute invasive form of fungal rhinosinusitis (FRS) caused by *Zygomycetes* in an immunosuppressed host [7]. *Aspergillus* can also cause acute invasive rhinosinusitis (McGill in 1980) [8]. Hora, in 1965, categorized fungal rhinosinusitis in two categories, namely invasive (osseous erosion and extension into tissue) and non-invasive (similar to chronic bacterial sinusitis) [9]. The understanding of both these categories progressively became clearer with the description of chronic granulomatous sinusitis in patients in the Sudan by Milosev in 1969 and fungal ball by Fimby and Begg in 1972 [10,11]. However, the pathology of fungal rhinosinusitis in some patients could not be explained, as allergic inflammation was a predominant feature in those lesions. Safirstein first coined the term “allergic *Aspergillus* sinusitis” in a patient to explain simultaneous involvement of lung and sinuses with similar pathology [12]. Subsequently in 1981, Millar described sinus symptoms with allergic pathology in five patients, though simultaneous history of allergic bronchopulmonary aspergillosis (ABPA) was seen in only one patient [13]. He coined the term “allergic aspergillosis of paranasal sinuses” as the mucus from sinuses of these patients histologically simulated the mucus plugs expectorated by ABPA patients and patients demonstrating a type I hypersensitivity reaction to *A. fumigatus*. In 1983, Katzenstein analyzed the sinus mucus material of 119 samples, of which nine samples were noted to have “allergic mucin” composed of mucin, eosinophils and Charcot Leyden crystals [14]. *Aspergillus* hyphae were detected in seven of those samples (mostly from young adult patients) simulating ABPA and leading to coinage of “allergic *Aspergillus* sinusitis (AAS).” This represented the fourth type of sinus aspergillosis described at that time following fulminant, indolent and localized non-invasive fungal ball (mycetoma)-like varieties [14,15,16]. Manning et al. (1989) reported the findings of AAS in six pediatric patients aged 8–16 years, four of whom presented with facial deformity [15]. However, the term AAS was changed to “allergic fungal sinusitis” when etiologic agents other than *Aspergillus* spp. (dematiaceous group including *Bipolaris* spp., *Alternaria* spp., *Curvularia* spp.) were identified [2,17,18,19]. The term “allergic fungal rhinosinusitis” was introduced by Robson et al. in 1989 to address the type of polypoid chronic rhinosinusitis where the patient had type I hypersensitivity, viscid allergic mucin and fungal hyphae in the sinuses [18,20]. Further, fungal hyphae were found to be missing in allergic mucin of some cases [17,21]. In 1994, Cody et al. suggested the term “AFS-like syndrome” for such cases [21]. Ferguson in 2000, coined the term “eosinophilic mucin rhinosinusitis (EMRS)” to describe those cases. However, there were some patients who, despite being non-atopic, developed similar symptoms. Ponikau et al. used novel diagnostic techniques for detecting fungi in mucin and concluded that most of chronic rhinosinusitis (CRS) cases were due to hypersensitivity to fungi, and hyphae were always detected in nasal secretions of those cases [14,22]. They gave a new term “eosinophilic fungal rhinosinusitis (EFRS)” to describe the patients with FRS with predominant eosinophil presence in sinus mucin. In the late 1990s, invasive FRS was categorized into fulminant, chronic and granulomatous forms by DeShazo et al. [23]. The non-invasive forms of FRS were categorized into saprophytic colonization, fungal ball and fungus-related eosinophilic rhinosinusitis (including AFRS) [24,25].

## 3. Classification

There is no consensus for the classification of FRS. A working group on “Fungal sinusitis” under International Society for Human and Animal Mycology undertook a workshop in 2009 and proposed the following classification [25]. Broadly, FRS was classified into invasive and non-invasive disease depending on invasion by fungi across nasal and sinus mucous membrane.

### 3.1. The Invasive Form Includes

- Acute (fulminant, necrotizing) FRS: This type is commonly seen in immunosuppressed patients (hematological malignancy, diabetes mellitus, transplant and on immunosuppressive drug) with history of less than 4 weeks. It is characterized by vascular invasion by fungal hyphae, necrotizing reaction with abundant hyphae. Occasionally bland necrosis is seen [23,26,27,28]. It is most commonly caused by fungi under *Mucorales* or *Aspergillus* species [1].
- Granulomatous invasive FRS: This form of FRS is seen in immunocompetent patients from tropical regions from Sudan to India [22,29,30]. The lesion typically presents with granuloma and sparse *A. flavus* hyphae with or without foreign body or giant cells. The duration of illness is more than 12 weeks and affects cheek, nose, orbit and paranasal sinuses with predominant proptosis.
- Chronic invasive FRS: This condition is seen in mildly immunosuppressed patients (diabetes, steroid therapy) and lasts for more than 12 weeks with progression at a relatively slow pace. It affects ethmoid and sphenoid sinuses commonly. Histologically, it presents with abundant fungal hyphae (commonly *A. fumigatus*), mixed inflammatory reaction, and occasional vascular invasion. The disease spreads to cheek; orbit-like chronic granulomatous type [23,29,31].

### 3.2. The Non-Invasive Fungal Rhinosinusitis (FRS) Comprises of Following Categories

- Fungal colonization: It is an asymptomatic saprobic colonization of nasal cavity or sinuses by fungi in immunocompetent hosts often after local surgery. It usually follows benign course [25].
- Fungal ball (previously known as sinus aspergilloma/mycetoma): It is defined as accumulation of dense conglomerated fungal hyphae in sinuses without invasion [32]. This condition generally affects older, immunocompetent patients (average age 64 years). Most commonly, it represents maxillary sinus colonization (followed by sphenoid sinus) by fungi with poor inflammatory reaction, often seen in adult immunocompetent females of southern France [32]. It is characterized by sinus opacification, cheesy discharge, chronic inflammatory reaction without any tissue invasion by fungi. Bone erosion is reported in 4%–17% patients. The exact pathogenesis of the condition is unclear although aerogenic and iatrogenic pathway theories are proposed [32]. According to aerogenic theory, a high burden of fungal spores make their way into sinuses through ostia while iatrogenic or odontogenic pathway is secondary to any dental procedure which causes formation of oro-antral communication. Upon microscopic examination, tightly packed hyphae are observed in alternating dense and less dense zones similar to concentric layers of onion skin which are surrounded by a dense inflammatory exudate of predominantly neutrophils. The diagnosis of fungal ball should be highly suspected in a patient of recurrent unilateral sinusitis refractory to treatment supported by CT findings of opacified sinus with central metal dense spots and microbiological and histopathological features. The isolation of fungi may fail sometime; diagnosis depends on microscopy and histopathology in those cases.
- Eosinophil-related FRS: This category suffers from confusion in defining three entities (AFRS, EMRS and EFRS) as distinct varieties.
  ➢ AFRS: It is characterized by nasal polyposis, type I (raised IgE) and possibly type III hypersensitivity reaction, production of allergic mucin with abundant eosinophils and non-invading fungal hyphae [25]. The fungi behave as allergens in atopic host causing inflammation of sinuses thereby obstructing the sinus ostia hampering drainage [25,33,34]. Occasionally, patients with recurrent AFRS may not have nasal polyps due to previous surgery though eosinophilic mucin and hyphae are present. DeShazo removed the criterion type I hypersensitivity in defining AFRS, as some researchers did not find immediate hypersensitivity in all patients with AFRS [35].
  ➢ EMRS: EMRS is described as a distinct entity by Ferguson [26]. It represents a systemic immune dysregulation where fungal hyphae do not play any role and are not detected in the eosinophilic mucin. It occurs in patients with asthma, aspirin sensitivity and IgG1 deficiency and is generally bilateral [25,26]. She proposed four types of eosinophil-related FRS: allergic fungal rhinosinusitis, non-allergic fungal eosinophilic rhinosinusitis, super antigen-induced eosinophilic rhinosinusitis, and aspirin-exacerbated eosinophilic rhinosinusitis [26]. The important features that distinguish EMRS from AFRS include age (young in AFRS, old in EMRS); nasal obstruction (100% in AFRS, one-third cases of EMRS); laterality (unilateral or bilateral in AFRS, bilateral in nearly all cases of EMRS); orbital involvement (common in AFRS); total IgE levels (raised in AFRS); fungal hyphae demonstration (absent in EMRS) and expression of genes for cathepsin B, sialyltransferase 1, GM2 ganglioside-activation protein and S100 calcium binding protein (absent in AFRS) [3].
  ➢ EFRS: Ponikau et al. described this entity to characterize the patients with FRS having fungal hyphae embedded in eosinophilic mucin with or without evidence of type I hypersensitivity [22]. His group even claimed that all cases of chronic rhinosinusitis are due to fungi as etiology. Braun et al. and Polzehl et al. supported the hypothesis by demonstrating fungi in sinuses of all cases of chronic rhinosinusitis using sensitive techniques, even without atopy [36,37]. They claimed that certain fungi might be able to mount eosinophilic immune response in the absence of atopy, which was further supported by the in vitro observation of elicitation of Th1 and Th2 responses by non-atopic CRS patients in response to fungal (*Alternaria* species) exposure [38].

## 4. Controversies: Where Do We Stand?

Although acute rhinosinusitis is well categorized, the classification of chronic rhinosinusitis is still subject to controversy. Fungal rhinosinusitis, a subset of CRS, faces differential opinion in being recognized as an infection or an inflammatory process. The most controversy exists in the eosinophilic fungal RS group. The role of fungi in causing CRS has continued to raise debates since 1999 when Ponikau et al. suggested fungi to be etiological agents in most cases of CRS [22]. They demonstrated this by isolating fungi in 96% patients of CRS with <25% demonstrating atopy (disputing the type I hypersensitivity theory in causation of the disease) and proposed the term “EFRS” to replace AFRS. However, 100% of healthy volunteers also demonstrate fungal hyphae on nasal mucosa. The authors had utilized highly sensitive diagnostic techniques to demonstrate fungal proteins in sinus mucus. They further attempted to confirm their hypothesis by demonstrating significantly accentuated Th1/Th2 responses, when peripheral blood mononuclear cells (PBMCs) from CRS patients were exposed to common ubiquitous fungi (*Alternaria* species). They also demonstrated clinical improvement in patients taking antifungal treatment enrolled in uncontrolled trials [39]. They finally concluded that chronic eosinophilic response in CRS might be attributed to abnormal immune and inflammatory responses to fungi and proposed antifungal treatment for all CRS cases [40,41]. However, clinical trials (intranasal amphotericin B) performed by others failed to produce significant outcome of CRS cases contrary to the claims by Ponikau et al. [41,42]. DeShazo et al. also opposed the above hypothesis by claiming low specificity of diagnostic methods used by Ponikau et al. and considered AFRS to be a unique entity among CRS [40]. The confusion further increased when Ferguson introduced the term “EMRS” to designate the cases where eosinophilic mucin lacked fungal hyphae, which rendered antifungal and immunotherapy ineffective in these cases [26]. However, presence or absence of fungi in eosinophilic mucin depends on technique used. Sensitive techniques like chitin staining and PCR could improve demonstration of fungi in those eosinophilic mucin [37,43,44,45]. Therefore, in many cases, rarity of fungal hyphae may lead to mislabeling of AFRS as EMRS. An overlap of clinical, radiological, and immunological features among AFRS, EFRS, and EMRS cases was reported though separate management protocols proposed for each entity [46,47].

The definition of AFRS was further challenged, when cases with histologically proven tissue invasion were described [48,49]. It may reflect the possibility of coexistence of chronic granulomatous variety and AFRS in the same patient or continuum of the disease from AFRS to chronic granulomatous stage [50,51]. Another view proposes that AFRS cases may have a progressive spectrum with non-invasive disease progressing to the invasive stage due to change in host immune status [52,53,54]. Further, the term “chronic destructive but non-invasive FRS” introduced by Rowe-Jones and Moore-Gillon in 1994 may relate to AFRS owing to its chronic course, erosive imaging features, requirement of surgical management, and prolonged follow up [55]. However, it differs from AFRS in terms of histopathological appearance, immune status of host, and management. Upon thorough examination, many of their cases have turned out to be those of AFRS [24,56]. AFRS also needs to be differentiated from chronic rhinosinusitis with nasal polyp (CRSwNP) where patients tend to be non-atopic Caucasians, in an older age group, with higher socioeconomic status, lower IgE levels, and lower Lund Mackay score, and generally affected by *Alternaria* species and *Cladosporium* species [57].

The categorization thus appears to be complex. An attempt to resolve the controversies regarding FRS was initiated by a working group on “Fungal sinusitis” under ISHAM in 2009 by organizing a workshop. They broadly categorized eosinophil-mediated diseases into fungal (AFRS, EFRS and some aspirin-exacerbated RS) and non-fungal (AFRS-like group with fungal-specific IgE, EMRS group, aspirin-exacerbated rhinosinusitis) forms [24,25]. They supported the term “eosinophilic mucin” instead of allergic mucin, as allergy might not be present in all cases. They concluded that the etiological role of fungi in all CRS cases, atopy in causing eosinophilic disease, and need of antifungal therapy lacked enough evidence [25,42]. They also highlighted the need of better definitions for AFRS, EFRS, and EMRS.

Many authors have attempted to elucidate the role of fungi in CRS [39,58,59,60,61]. It is true that sensitive methods are capable of detecting fungal spores in the nasal mucosa that are prevalent in air. However, the role of fungus or relative amount of spore in the environment that makes the susceptible population at risk is not yet clear. It is believed that excreted proteases from colonizing fungal spores may breach the epithelial integrity exposing the mucosa to fungal hyphae [62]. Recently, fungus has been noted as a constituent of biofilms in a significant proportion of patients with CRS, although its presence as a contributor or an inert member is yet to be described [63,64]. A clear understanding of the role of fungi may help in therapy of CRS patients. The double-blinded and randomized studies investigating the role of topical antifungals and systemic antifungals failed to show any positive response [65,66,67,68,69,70,71]. Some of the workers believe that fungi may have a disease-modifying role in the dysregulated immune system of the CRS host rather than a causative role. It might also be possible that entrapped hyphae within mucus or biofilms in some of these cases may increase the already present immune and inflammatory response [42].

## 5. Epidemiology

Rhinosinusitis affects about 20% of the population once in a lifetime [1]. In the US, 4% of adults are affected annually [72]. The prevalence of FRS is difficult to assess due to controversy about its definition. The prevalence would be very high if we agree with Ponikau et al.’s proposition that all CRS cases are due to fungi [22]. Overall, CRS affects 1%–1.5%, 11% and 12.5% of the population in North India (rural), the European Union and the US, respectively [73,74,75]. Currently, AFRS is responsible for 7%–12% of CRS cases undergoing sinus surgery [76]. Of the total cases of CRS, FRS is observed in 27.2% cases (1.1 persons per 1000 population) in India indicating high burden of FRS cases in rural northern India [73]. Climate possibly plays an important role in the considerably high prevalence of FRS cases in India, Sudan, and Pakistan [1].

■ Geographical variation: AFRS is reported in areas with warm, dry and humid climate [3]. The high prevalence of the disease is noted in India, North Africa, the Middle East and southeastern and southwestern parts of the US (especially Mississippi basin) [46,77,78,79,80,81,82,83,84,85]. Northern states of the US have a lower frequency of 0.4%, while Southern states reported ≥10% [79]. AFRS constitutes the highest number of cases of CRS in India accounting for 56%–79% of cases [51,73,84,86]. AFRS cases are also reported from Australia, Malaysia, and Thailand [18,87,88].
■ Seasonal variation: The study from rural northern India reported a correlation of high incidence of FRS with wheat-harvesting season in winter months, when fungal spore count in the air increases due to wheat thrashing [73].
■ Host factors: AFRS is observed commonly in young adult males from rural areas attributed to their work in the fields in warm climates, thus predisposing them to nasal mucosal injury and fungal colonization [73,78]. Other predisposing factors include African-American origin, structural anomalies, and low socioeconomic status. Bony erosion is 15 times more common in African-Americans with higher rate of intraorbital and intracranial extension of the lesion [89,90,91,92]. While Ghegan et al. failed to observe any correlation between bony erosion and low socioeconomic status, other studies have found a significant correlation between the bony erosion and inhabitants of low-income countries with poor housing conditions [3,92,93]. Patients with intracranial and intraorbital extension of the disease were also found to be residents of rural areas where primary healthcare was poor and patients reported to hospitals only in the later stages of the disease [94]. HLA studies have shown higher association of AFRS with DQB1*301 and *302 [95]. Other host factors include atopy, asthma, and aspirin sensitivity [26,96].
■ Agent factors: Manning and Holman reported isolation of 87% dematiaceous fungi and 13% *Aspergillus* species from patients with AFRS [97]. However, Montone et al. reported higher (34%) isolation of *Aspergillus* than dematiaceous (30%) fungi [46,98]. *Aspergillus flavus* is the most common isolate (upto 96%) from patients with AFRS from India and Sudan [73,78,80,81,82,84,85,87]. Similarly, *A. flavus* was isolated from >50% patients with AFRS in the Middle East [83].

## 6. Clinical Presentation

**Figure 1:**
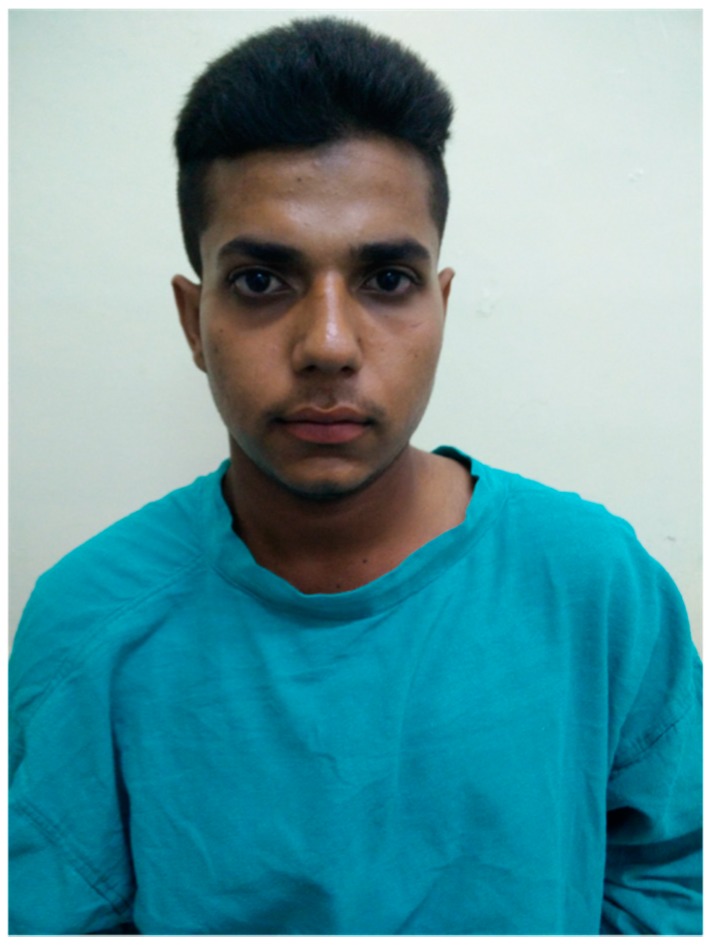
An 18-year-old male patient with allergic fungal rhinosinusitis (AFRS). The patient presented with left cheek swelling and right proptosis.

A patient with AFRS is usually an immunocompetent atopic young adult or an adolescent, and less commonly a child, though the disease has been found in all ages [3]. The patient complains of unilateral or bilateral symptoms of chronic rhinosinusitis with nasal polyposis and viscid, dark mucoid discharge with greenish black nasal casts not responding to medical or surgical therapy aimed at combating bacterial etiology [19,99]. Children usually present with unilateral disease (70% cases) while only 37% adults have one-sided presentation [76]. Patro et al. observed AFRS in children to be more aggressive with higher fungal load and less response to treatment as compared to adults [100]. Complications of AFRS include visual disturbances, proptosis, telecanthus, facial deformity, neuropathies or intracranial abscess (Figure 1) [57,101,102,103,104]. Bony erosion is observed in the majority of cases belonging to a young age group and being African-American [90,99,105,106]. It probably occurs due to blockage of ostia of the sinuses by polyposis leading to expansion of sinuses [105]. Commonly, the ethmoid sinus is affected with lesion extending to orbit (especially lamina papyracea) and the anterior cranial fossa [90,105]. In general, 66% of AFRS patients have a history of allergic rhinitis, 90% demonstrate increased specific IgE to one or more fungi, and around 50% suffer from asthma [107].

## 7. Pathogenesis and Immunology: Recent Concepts

**Figure 2:**
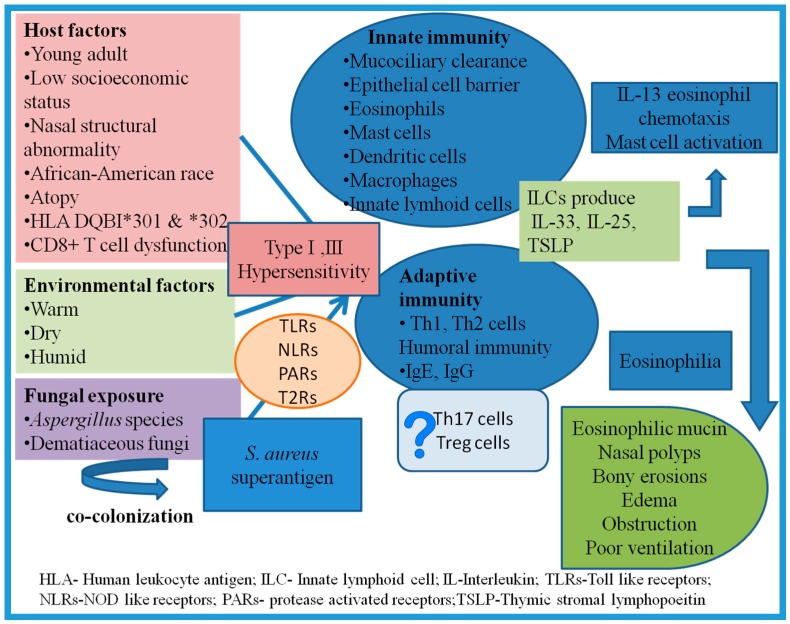
Complex interplay of various factors in etiopathogenesis of AFRS.

The pathogenesis of AFRS is unclear, though it has been evolving in recent studies. It is considered to be a complex interplay of IgE-mediated systemic/local hypersensitivity to fungal antigens, host-defense mechanisms, and possibly superantigens (Figure 2) [22,97,108,109,110,111]. The role of fungi in initiating or maintaining the disease process remains controversial. The initiation of disease requires a genetically susceptible host, who is resident of a humid, warm climate and exposed to fungal allergens.

### 7.1. Role of Atopy

A role of both systemic and local IgE hypersensitivity is proposed in etiopathogenesis of AFRS. The earliest reports suggested the simulation of this condition to that of ABPA (nasal polyposis, crust formation, eosinophilia and positive sinus fungal cultures (*Aspergillus*), increase in total and fungal-specific IgE) and attributed it to type I and probably type III hypersensitivity [12]. Manning et al. and Stewart et al. supported this immunologic mechanism and suggested the role of fungal antigens (*Bipolaris*) in eliciting fungal-specific IgE and IgG antibodies in blood and eosinophilic inflammatory infiltrate [97,112]. Feger et al. confirmed the association of AFRS with allergy by demonstrating significant increase of eosinophil chemo-attractant protein (ECP) in AFRS patients in comparison to control population [113]. The role of fungi and inflammatory meditators (IL-5, eotaxin) in eosinophil degranulation were shown in in vitro studies. The fungi and eosinophil interaction vary with inciting fungal agent. Inoue et al. demonstrated eosinophil degranulation on interaction with *Alternaria* species, but no or only a mute response during interaction with *A. flavus,* whereas Kale et al. demonstrated in their patients eosinophil degranulation and high release of MBP upon stimulation with *A. flavus* and abscence or mute response with *A. alternata* [47]. This dichotomy may be attributed to the higher prevalence of *A. flavus* as causative agent of AFRS in India in contrast to the Western world where *A. alternata* predominates. They concluded that the variation in patient population and responsible fungal agents in different geographic regions might be responsible for the contrasting results.

### 7.2. Exposure to Antigens

The fungi are ubiquitously present in the environment, and sinonasal mucosa is continuously exposed to fungi their antigens. The local microbiome is known to harbor a variety of bacteria, fungi and probably viruses [114]. The fungal agents are considered important colonizers in cases of CRSwNP rather than without polyps [114]. β-d-glucan in cell wall of fungi is considered one of the important antigens initiating the inflammatory cascade in a susceptible host [47]. An 18 kDa pan-fungal allergen present within eosinophilic mucin probably combines with host receptors leading to activation of signal transduction pathways [115]. However, enhanced T-cell response in EMRS patients in the absence of fungal antigens also suggested the role of other non-allergic antigens in immune system stimulation [111].

### 7.3. Innate Immune Response

(a) Mucociliary clearance: Epithelial lining of respiratory tract possesses cilia, which wash out the unwanted particles and pathogens by their rhythmic movements [116]. Their function is further aided by mucus production. The upper layer of this airway surface liquid comprises of antimicrobial rich mucus gel while the lower layer is a thin fluid surrounding the cilia supporting their rapid movement [117]. The rhythmic beating of these cilia transports thick mucus layer thereby flushing out the debris from sinonasal cavity. Acquired ciliary dysfunction due to environmental/ microbial toxin is observed in response to *A. fumigatus* and *S. aureus* [118,119]. The resulting mucostasis and hypoxia affects the ion transport and provokes polyp formation [120,121]. The level of an epithelial anion transporter, pendrin, is increased in nasal polyps and is linked to IL-4, IL-13 and IL-17A production, although its role in mucociliary clearance and pathogenesis of CRSwNP is not clearly elucidated [122,123,124]. Sheshadri et al. also noticed a significantly high level of Muc5AC (causes increased mucus production) in nasal polyps of patients with CRSwNP as compared to those without nasal polyposis or healthy controls [125]. Another bitter taste receptors type 2 (T2R) expressed by ciliated epithelial cells are being explored as an important part of first line defence mechanism [126,127,128,129]. They are linked to enhanced mucociliary clearance, nitric oxide production and release of antimicrobial peptides. 

(b) Epithelial cell barrier: The damage caused by the inhaled allergens is prevented by the physical barrier of epithelial cells comprised of tight junctions, adherens junctions and desmosomes [130]. Patients with CRSwNP have demonstrated significant decrease in number of tight junction proteins (occluding-1, zonula occludens-1 and claudin) and desmosomal proteins (DSG2 and DSG3) in comparison to healthy controls [75,131]. Den Beste et al. showed a 41% decrease in transepithelial resistance in AFRS patients highlighting the reduction in tight junction proteins and increase in leaky junction proteins [132]. LEKT1, an epithelial protein possessing protease inhibiting activity is also diminished significantly in CRSwNP increasing vulnerability to protease activity of fungi [75]. Some of the other abnormalities observed in CRS patients include goblet cell hyperplasia leading to increased mucus production, variation in ion transport, basal cell proliferation, acanthosis and acantholysis. Epithelial barrier dysfunction in CRSwNP patients is hypothesized to be attributed to either intrinsic defects or to increased levels of oncostatin M (member of IL-6 family), an inducer of tissue permeability [133]. Additionally, many bacteria (especially *S. aureus*) and fungi are capable of producing molecules disrupting the zona occludens-1 of human nasal epithelial cells [130]. Microbe associated proteases also have the property of cleaving junctional proteins and inducing changes in epithelium through protease-activated receptors (PAR-2) [134,135].

(c) Pattern recognition receptors: These receptors expressed on sinonasal tissue include TLRs, PARs, NLRs and T2Rs, which recognize PAMPs [136,137]. All 10 types of TLRs are expressed in the sinonasal epithelial cells but their expression varies in CRS. While TLR2, TLR4 and TLR7 are noted in high level in CRS patients, the role of TLR9 is not clear as different studies have shown both downregulation and upregulation [138,139]. Of the four types of PARs highly expressed in respiratory tract, PAR-2 plays an important role in allergic airway inflammation [140]. Ebert et al. noticed increased expression of PARs especially PAR3 in AFRS patients versus controls [141]. Their stimulation by fungal proteases causes eosinophilic infiltration and airway hyperactivity with release of cytokines accentuating/enhancing Th2 response [142,143]. The increase in neutrophils in CRS tissue also occurs in response to IL-8 secreted by PAR-2 stimulation [75]. NLRs associated with fungal infection (NOD1, NOD2, NALP3, NLRC4) are expressed in tonsils, adenoids, lung, nasal mucosa, nasal epithelial cells, lung epithelial cells and neutrophils [144,145]. They are known to play a role in producing inflammatory cytokines and antimicrobial peptides production [144]. The major components of fungal cell wall, β-glucans and mannan, are recognized by C-type lectins including Dectin-1 and Dectin-2 leading to stimulation of host immune response [146]. The induction of IL-6 and IL-8 has been observed in in vitro studies on stimulation with β-glucans. They are also believed to promote allergic sensitization in lung triggered by β-glucans [147,148]. Like many chronic inflammatory diseases, AFRS follows either MAPK signalling pathway or NF-κB signalling pathway [149,150]. MAPK family comprises of three major proteins: extracellular signal-regulated kinase (ERK), p38 MAPK (including p38α, p38β, p38γ, and p38δ), and c-Jun *N*-terminal kinases (JNK including JNK1, JNK2, and JNK3) [151]. MAPK works further by activation of NF-κβ pathway like upregulation of COX-2 expression in CRS [152]. p38 MAPK pathway controls the expression of pro-inflammatory cytokines (such as TNF-α, IL-1, IL-2, IL-6, IL-7) and matrix metalloproteases (MMPs such as MMP-2, MMP-9, and MMP-13), leukocyte adhesion, chemotaxis, oxidative burst (inducible nitric-oxide synthase , iNOS)and degranulation [151,153,154,155]. Therefore, the activation of pattern recognition receptors (PRRs) by allergens initiates the intracellular signalling, activation of NF-κB, which subsequently upregulate the expression of genes involved in immune response including cytokines, chemokines, growth factors and antimicrobial peptides [156]. Further, JAK-STAT1 signal is shown to be inhibited by fungal extracts which causes suppression of Th1 and favours Th2 pathway [157]. The expression of IL-22 and STAT3 function responsible for mucoid immune regulation, host defence and post traumatic regeneration is diminished in CRS cases [158,159,160].

(d) Secretory products of epithelial cells: The pseudo-stratified ciliated respiratory epithelial cells besides their physical barrier role, produce a wide range of antimicrobial factors including antibodies, defensins, complement, chemokines (IL-8, MCP-1), surfactant proteins, lysozyme, lactoferrin, antitrypsin, S100 proteins which act against microbes [161]. Defensins are responsible for formation of pores in fungal and bacterial cells. Collectins like surfactants, C-reactive protein and MBL play a role in recognizing PAMPs leading to their early clearance [162]. The role of lysozyme in CRS is debatable as studies have shown both increased and decreased levels in these patients [163,164,165]. Although, lactoferrin chelates iron and produces iron deficient environment for fungi and bacteria affecting their metabolism, its levels are noticed to be decreased in CRSwNP patients [75,166,167]. Low levels of SPLUNC-1, S100 A7 (psoriasin), S100 A8/A9 (calprotectin), defensins and LL-37 observed in CRSwNP patients reflects the diminished antimicrobial activity in their sinonasal mucosa [165,168,169,170]. Other molecules produced by epithelial cells include reactive oxygen and nitrogen species like lactoperoxidase, NADPH oxidase and nitric oxide [130,171]. The cytokines, IL-25, IL-33 and thymic stromal lymphopoetin (TSLP) released by the epithelial cells polarize the immune response towards Th2 type. The ST2 receptors for IL-33 are present on mast cells, eosinophils, T cells and innate lymphoid cells (ILC-1,2,3) [172]. IL-25 (member of IL-17 family) and IL-33 stimulate the ILCs to produce IL-13 and eosinophil chemotaxis [173]. Shaw et al. observed significant increase in ILC2s (associated with Th2 cytokines) in nasal polyps [172,174]. IL-22 has an allergy suppressive effect as noted in various studies probably by decreasing expression of IL-25 [175,176]. High concentration of IL-17 and myeloperoxidase are also observed in polyps [75]. Mast-cell activation occurs in response to increased TSLP [177]. TSLP promotes Th2 response and its increased activity has been noticed in nasal polyps of CRSwNP patients in comparison to healthy subjects [177]. Recently, a significant increase in P-glycoprotein (P-gp) was noticed in CRSwNP patients as compared to other CRS and is associated with secretion of IL-5, TSLP, IL-6 and GM-CSF skewing the response towards Th2 type [178]. These observations suggest an important role of IL-33, IL-25 and TSLP in immunopathogenesis of AFRS. Chemokines secreted by epithelial cells include eotaxin-1 (CCL11), eotaxin-2 (CCL24) and eotaxin-3 (CCL26) which have been demonstrated in increased numbers in nasal polyps as compared to healthy controls [130,179,180,181].

(e) Macrophages: Macrophages, an integral part of innate immune system comprise of M1 and M2 types based on their production of Th1 (protective in nature; secrete pro-inflammatory cytokines, such as IL-1β, IL-12, IL-23 and tumor necrosis factor (TNF), as well as high levels of effector molecules, including nitric oxide) or Th2 response (immunosuppressive in nature; increased expression of non-opsonic receptors like mannose receptor, scavenger receptor-1, CD163, Trem-2) respectively [182]. Of these, M2 macrophages or alternatively activated macrophages are believed to play a role in allergic diseases [183,184]. Their presence in CRSwNP patients has been associated with release of CCL18 like chemokines favoring Th2 response [75].

(f) Dendritic cells: These are the antigen-capturing cells capable of activating both innate and adaptive arms of the immune system causing T-cell differentiation towards Th2 subset by releasing IL-4, IL-5 and IL-13, in turn causing B-cell switching to IgE isotype and release of fungal-specific IgG, IgE and eosinophil accumulation [185]. Ayers et al. showed an increased number of local dendritic cells in AFRS versus control subjects [186]. The role of vitamin D3 in immunopathology of AFRS was evaluated by Mulligan et al. [187]. They noticed lower vitamin D3 in AFRS patients that inversely correlated with increased number of mature dendritic cells and bony erosions in CT scan. Vitamin D3 acts as a disease-modifying factor in CRSwNP cases [188]. 

(g) Other cells: Eosinophils, basophils, mast cells and innate lymphoid cells (ILCs) (already mentioned previously) release cytokines favouring Th2 response (IL-5, IL-13). Specialized mast cells secreting chymase, tryptase and carboxypeptidase A3 identified in CRSwNP cases are hypothesized to produce excess mucus [130]. 

In a nutshell, it is proposed that initially innate immune cells (eosinophils, mast cells, ILCs, dendritic cells, macrophages) accumulate when fungus and epithelial cells interact, leading to production of cytokines causing activation of robust adaptive immunity [189]. 

### 7.4. Adaptive Immune System

Th2 polarization occurs due to orchestration of M2 macrophages, TSLP, IL-4, IL-25 and IL-33. Th2 cells secrete IL-4, IL-5, IL-9 and IL-13 cytokines leading to IgE secretion, eosinophil chemotaxis causing chronic inflammation. Increased levels of IL-5 locally within nasal polyps have been noted in patients of CRSwNP [190,191]. IL-5 causes maturation of eosinophils in bone marrow and aids in their release into the blood [192]. The production of IL-5 follows autocrine secretion pattern thereby maintaining localized eosinophilic inflammation. IL-13 causes eosinophil chemotaxis class switching in B-cell (IgE phenotype), mucus hypersecretion and airway hyperresponsiveness in allergic diseases [193]. A higher ratio of CD4+ to CD8+ T cells is observed in CRSwNP than without polyposis [130]. Pant et al. observed failure of CD8+ T cells present in sinuses of AFRS and EMRS patients to proliferate and express CD25 (activation marker) in response to fungal antigen exposure (both *Alternaria alternata* and *A. fumigatus*) as compared to healthy controls [108]. They hypothesized that dysfunctional CD8+ T cells in AFRS patients may be responsible for ineffective clearance of fungal elements from their sinuses thereby predisposing the individuals to AFRS. The defect in CD8+ T cell increases susceptibility to other form of aspergillosis as well [108]. Despite the defect in CD8+ T cells, fungal-specific IgG3 is believed to play a protective role in AFRS and EMRS patients [108,111]. Role of Treg cells in pathogenesis is still controversial. Lam et al. demonstrated suppressor function of Treg cells creating imbalance between proinflammatory and anti-inflammatory factors in CRSwNP patients, while Pant et al. noted increased number of T reg cells in such patients [143]. Interestingly, an important difference is noted in inflammatory patterns of Caucasians and Asian people. European and American studies have shown predominance of Th2 cytokines (IL-4, IL-5, IL-13) in CRSwNP patients, which further invite eosinophils, basophils and mast cells [130]. However, on the contrary, Asian studies have demonstrated Th1 response in majority of patients with increase in IFN-γ and low IL-5 levels which may be explained by yet unknown genetic factors [194,195]. The predominant effector cell in such patients is Th17 cell. Additionally, an increase in neutrophil number and decrease in levels of eosinophils, eotaxin and ECP is observed in Asian patients. 

Apart from T cells, the numbers of naive B cells and activated plasma cells are elevated in response to CXCL13 and CXCL12 in nasal polyps of CRSwNP [196,197,198]. A significant rise in levels of IgA, IgE and IgG are observed in nasal tissue of CRSwNP [75]. Collins et al. (2004) suggested localized (within nose and sinuses) type I hypersensitivity rather than systemic hypersensitivity in pathogenesis of AFRS by illustrating the higher presence (71%) of fungal-specific IgE in sinus mucosa [110]. This hypothesis may explain why all patients with AFRS do not exhibit signs of systemic allergy. They demonstrated presence of fungus-specific IgE within the eosinophilic mucin of AFRS patients thereby confirming the role of fungal allergy. Chang and Fang showed presence of *Aspergillus*-specific IgE in maxillary sinus tissue of 87.5% of AFRS patients despite absence of any serum IgE response [199]. Recently, Wise et al. and Ahn et al. demonstrated highest localization of IgE in subepithelium of inferior turbinates and sinuses in AFRS patients as compared to the controls [200,201]. The detection of IgE encoding transcripts in sinus mucosa of patients further emphasizes the need for research in this area [202]. It is concluded that the B cells cause local rise of antibodies IgG, IgA, IgE and IgM without any parallel increase in peripheral blood levels thereby highlighting localized nature of inflammatory response. Antibodies specific to IgE against enterotoxin of *Staphylococcus aureus* have also been found in nasal polyps, which suggests the role of superantigens in etiopathogenesis of CRS. 

### 7.5. Role of Superantigens

The role of superantigen-induced chronic inflammation by polyclonal T-cell and B-cell activation in pathogenesis of AFRS was first noted by Schubert et al. [203]. *S. aureus* is frequently isolated (20%–30%) from CRS patients, but it is not clear whether it has some etiologic role or acts as a disease-modifying factor [189]. Nasal polyps generally are colonized by bacteria (upto 77% positive cultures) [204]. Clark et al. observed significantly higher colonization of *S. aureus* in AFRS vs. non-AFRS patients (63.2% vs. 24.1%) [205]. Elevated levels of serum-specific IgE to enterotoxin A and B along with fungal-specific IgE were demonstrated in AFRS patients [95,206]. The superantigens have the ability to activate up to 30% lymphocytes by serving as a bridge between antigen-presenting cells (APC) and lymphocytes-expressing specific TCR variable beta (Vβ) chains that bypass the normal path of antigen recognition, subsequently leading to tremendous cytokine-secretion favoring Th2 response [144,207]. They stimulate production of polyclonal IgE by B cells, which reinforce the Th2-cell activation and cause persistent inflammation [75]. However, a causal relationship is not yet established due to its ubiquitous presence [189]. It is proposed that these superantigens accentuate and skew the local eosinophilic response towards Th2 pathway promoting polypogenesis and persistent eosinophilic inflammation and are considered as disease modifiers rather than disease-causing agents [143,206]. In addition, these superantigens probably lower COX pathway causing an increase in PGD2 (skews towards Th2 pathway) and decrease in levels of PGE2 and its receptor, EP2, in nasal tissue of CRSwNP patients [75].

All the above-listed factors working in orchestration lead to complex tissue remodeling of nasal polyps and chronic inflammation. The role of TGF-β in polypogenesis is debatable as studies have shown contrasting results [208,209]. In addition, dysbalance between fibrin deposition and degradation has also been proposed for polyp growth [210,211].

Apart from immunologic mechanisms, local anatomical structure also plays an important role, which may explain unilaterality of the disease [34]. Fungi once trapped in nasal mucosa, stimulate the host immune system (IgG, IgE, IgA), which over a period of time leads to development of polyps, anomalous sinonasal structures, and bony erosions [1]. When the normal drainage pathway of sinuses is disturbed, viscid eosinophilic mucin accumulates, thereby raising the inflammatory markers leading to chronic inflammation [1].

## 8. Diagnosis

The diagnosis of AFRS is based on combination of clinical, radiological, microbiological and pathological findings. The earliest diagnostic criteria which is still widely accepted was formulated by Bent and Kuhn in 1994 [212]. The criteria included type I hypersensitivity, nasal polyposis, typical CT findings (as mentioned below), and eosinophilic mucin containing fungus without invasion across the mucous membrane. Later, minor criteria like asthma, Charcot Leyden crystals, eosinophilia, unilaterality of disease, fungal culture and bony erosion were added [213]. The criteria of type I hypersensitivity and typical CT findings are accepted for diagnosing AFRS by a European position paper on rhinosinusitis and nasal polyps, 2012 [214]. Various other criteria have also been proposed which have been refined eventually by working groups for defining different types of rhinosinusitis [215]. Loury et al. gave diagnostic criteria for AFRS in 1993 simulating Rosenberg’s criteria of ABPA [216]. It included eosinophilia, type 1 hypersensitivity, IgG to fungal antigens, elevated total IgE, nasal blockage, CT/MRI findings and histopathological description of allergic mucin. Cody et al. in 1994 modified the above criteria to only presence of allergic mucin and fungal hyphae or culture [21]. deShazo and Swain proposed in 1995 inclusion of sinusitis on X-ray, visual/pathological allergic mucin and fungal elements microscopically and/or culture, immunocompetency and lack of tissue invasion [35]. Saravannan et al. considered four important features for distinguishing AFRS from EMRS: type 1 hypersensitivity to fungi, CT findings, presence of allergic mucin with Charcot Leyden crystals and microscopic detection of fungi [46].

Various authors have attempted scoring of AFRS. Kupferberg and Bent categorized the patients postoperatively into stage 0 (no disease), stage I (allergic mucin and mucosal edema), stage II (allergic mucin and polypoid edema) and stage III (nasal polyps with or without fungal debris) [101]. Phillpott et al. considered this four-stage postoperative criteria ineffective as it included only one-sided sinus cavity which may give inaccurate staging [217]. They validated a novel ten-grade system, where each sinus cavity (maxillary, ethmoid, frontal and sphenoid) scored 0–9 for rising mucosal edema and a single point for fungal mucin, thereby providing the highest score of 40 for each nasal cavity. This score was found to be more descriptive along with providing information on response to therapy. Lund Mackay scoring was finalized in 1997 for staging of rhinosinusitis where a score was given to each sinus based on CT findings; 0 (normal), 1 (partial opacity) and 2 (complete opacity) with a total score of both sides ranging from 0 to 24 [218,219,220]. Opacification/development ratio (ODR) was proposed by Neto et al. for use in children whose sphenoid and frontal sinuses are not yet developed [221]. Wise et al. formulated a 24-point staging system in AFRS patients by adding bony erosions as separate entity [90]. They observed that males and African-Americans scored significantly higher than females and Caucasians in terms of bony erosions.

### 8.1. Imaging

**Figure 3:**
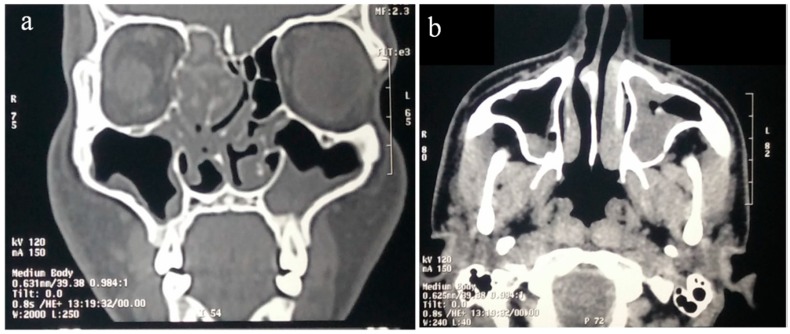
Coronal (**a**) and axial (**b**) computed tomography maxillofacial scan from the 18-year-old male patient with documented allergic fungal rhinosinusitis. There is opacification of left maxillary sinus and right ethmoid sinus with characteristic bony expansion and erosion.

CT scan is the initial investigation of choice as it shows typical findings in AFRS consisting of multiple sinus opacifications with central hyper-attenuation (central serpiginous or starry sky appearance), sinus mucocele, skull base erosions (56% of AFRS patients versus 5% of non AFRS patients) and remodeling with a “pushing border” at skull base (Figure 3) [20,89,90,222]. Proptosis with orbital erosion is observed in 50% of AFRS in the pediatric age group [223]. The characteristic features of AFRS include central low T1 and T2 void in sinuses which is due to presence of eosinophilic mucin (>28% protein concentration) surrounded by low T1 and high T2 signal intensity of inflamed mucosa enhanced by intravenous gadolinium contrast [91,99,222,224]. Occasionally, iso-intense or hypo-intense T1/T2 signal may be visible, which is caused by ferromagnetic elements [91]. Absence of signals on T2 imaging is due to higher protein and low free-water content in eosinophilic mucin together with calcium, iron, magnesium, and manganese [222,224].

X-ray of paranasal sinuses shows haziness of multiple sinuses, thickened mucosal lining and bony erosions. This modality is the least specific [225].

### 8.2. Microbiology

- Microscopy: The eosinophilic mucin and debris of sinus contents demonstrate fungal hyphae on direct KOH mount or more sensitive calcoflour white stain.
- Culture: Culture of sinus contents shows positive results in 10%–93% of AFRS cases [117,226,227]. However, growth of fungus in culture media does not always signify AFRS, as fungi are ubiquitous and may give false-positive results. Ponikau et al. demonstrated 100% positive-culture results in both patients and controls with an average of 2.3 organisms per host [22]. A negative culture does not rule out AFRS and a positive culture may represent environmental contamination. Thus, culture results act as mere supportive evidence for AFRS.
- Serology: Type I hypersensitivity to fungi is demonstrated by either ImmunoCAP or skin prick test, the former being more specific and having higher negative predictive value [228]. It is observed that AFRS patients possess high levels of specific IgE to multiple fungi which may aid in differentiating them from other CRS cases [112]. Total IgE in these patients is often more than 1000 IU/mL [99]. The role of fungal-specific IgG in diagnosis of AFRS is uncertain as it is also elevated in other varieties of AFRS. Fungal-specific precipitins may also be observed in 85% of AFRS patients [16]. However, the role of allergy is still questionable in AFRS. All patients may not display increased IgE levels or a positive skin test [94].
- Surface-enhanced laser desorption/ionization time-of-flight mass spectrometry (SELDI-TOF MS): It allows protein profiling of serum and identifies AFRS patients with sensitivity of 84% and specificity of 90% [229]. However, the routine application of this technique is not yet recommended.
- Molecular test: A PCR using ITS1/ITS2 performed directly on samples from CRS patients demonstrated sensitivity of 100% confirming its superiority over culture and also allows accurate identification by sequencing [230].

### 8.3. Pathology

**Figure 4:**
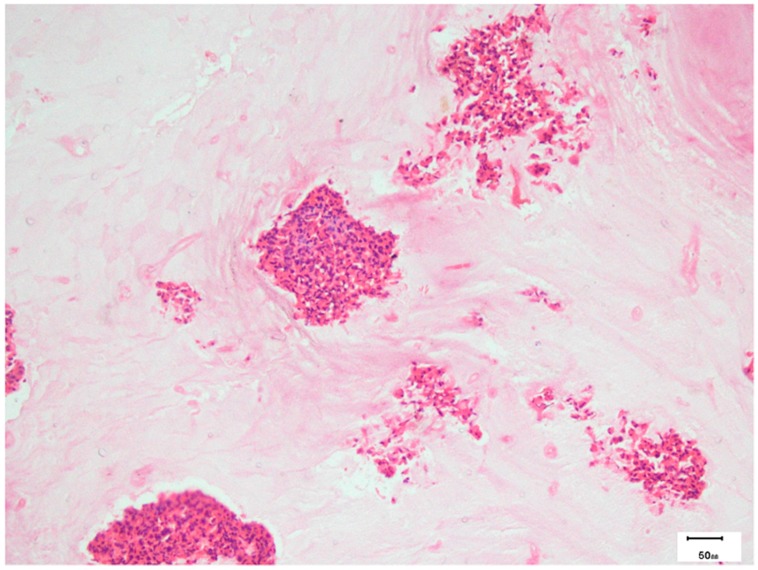
Photomicrograph showing alternate light and dark areas in the allergic mucin with eosinophilic clusters (hematoxylin and eosin stain) of the above patient.

**Figure 5:**
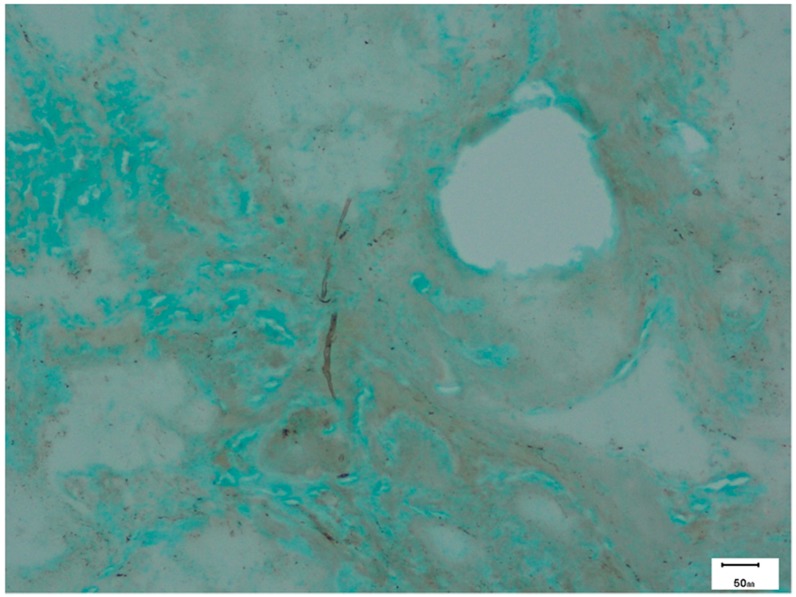
Photomicrograph showing occasional *Aspergillus* hyphae (Grocott’s methenamine silver stain) in the same patient.

Histopathology provides clear evidence of AFRS. Grossly, the eosinophilic mucin is viscid, tenacious, peanut butter-like and has a dark-greenish to brown color. Microscopically, hematoxylin and eosin (H&E) staining shows eosinophilic mucin in the form of onion laminations of eosinophils and their degradation products in the center surrounded by light-stained mucin and Charcot Leyden crystals (Figure 4) [109]. Polypoid mucosa is edematous with inflammatory mixture of lymphocytes, eosinophils, and plasma cells [51]. Routine H&E staining shows hyphae as a negative image and are detected in 67.5% of AFRS cases. The morphology of hyphae may be distorted, swollen, and have central pallor [51]. Special stains like periodic acid-Schiff (PAS) and Grocott’s methenamine silver stain (GMS) are required to demonstrate fungal hyphae (Figure 5). Immunofluorescence technique was used by Laury et al. to demonstrate increased levels of extracellular matrix protein periostin in sinus mucosa of AFRS patients [94].

## 9. Management

The understanding of management of AFRS is also evolving like pathogenesis and definition. The combination of surgical and medical therapy is important for management. The basic aim is to diminish the inflammatory trigger and subsequent inflammatory events.

### 9.1. Surgical

Earlier radical surgery was performed to remove the whole mucosa. Currently, endoscopic tissue-sparing (conservative) technique called functional endoscopic sinus surgery (FESS) has surpassed it as the surgery of choice [48,231]. The main goal of surgical therapy is to remove the antigenic stimulus from the sinuses, relieve the obstruction by nasal polypectomy, removing mucin, debris, and fungal elements to improve ventilation, restore mucociliary function, and provide easy access for further debridement or local therapy [232]. It aims to cure inflammatory disease of the sinuses by resecting the anatomical and inflammatory factors causing obstruction in the ostiomeatal unit while preserving the marginal mucosa, thereby avoiding radical surgery. The minimally invasive sinus surgery (MIST) includes the use of a shaver for improving precision. The technique involves uncinectomy, removal of the postero-medial wall of the agger nasi cells, opening of the bulla ethmoidalis, repositioning of the middle turbinate and removal of polyps and dilatation of sphenoid sinus access [233]. It is recommended to enlarge the maxillary sinus to the maximum possible width through the middle meatus in AFRS patients. AFRS is considered to have poor surgical outcome among all types of CRS. The FESS improves quality of life although revision surgery is required in 15%–20% patients [234]. The factors contributing to need of revision surgery are poor drainage of the frontal recess or the frontal sinus neo-ostium due to the presence of remains of the uncinate process and anterior ethmoid cells, a missed maxillary sinus ostium, a lateralized middle turbinate, scarring, osteoneogenesis, or recurrent polyposis [233]. The disease-specific measures and quality of life are predicted to be poor when the amount of mucosal eosinophilia is >10 eosinophils/high-power field during FESS procedure [235,236]. The patient is closely followed up and prescribed medical management to keep a check on disease recurrence and provide sufficient time for allowing normal mucosa to re-establish [94].

### 9.2. Medical Therapy

The medical management of AFRS lacks consensus among otolaryngologists. The major objective is to prevent recurrence.

- Saline irrigations: If given both pre- and postoperatively, the saline irrigations aid in softening and debriding thick mucoid secretions and improve mucociliary function of epithelium [94].
- Corticosteroids: Similar to surgical therapy, oral steroids are the mainstay of management of AFRS and have a significant role postoperatively in reducing recurrence and inflammatory markers, and ultimately improving the outcome in these patients. They may even obviate the need of revision surgery [107,189,234]. Gan et al. reviewed the available literature and found four studies (two level 2b and two level 4 studies), which looked into the benefits of oral steroids in AFRS and recommended the use of tapering doses of oral steroid [232]. The benefit of oral steroids in AFRS was first demonstrated in retrospective case series by Kupferberg et al. and Kuhn and Javer [237,238]. Woodworth et al. observed better SNOT-20 and nasal endoscopic scores and diminished levels of IL-3, IL-5, eotaxin, and monocyte chemoattractant protein-4 (MCP-4) when oral prednisolone was used [239]. Landsberg et al. demonstrated the radiologic and endoscopic benefits of preoperative administration of oral steroids in AFRS patients as compared to other CRSwNP cases, although the number of AFRS patients was low [240]. Their use in preoperative period helps in removing mechanical obstruction and that helps in viewing sinonasal anatomy during FESS [3]. Rupa et al. showed significant improvement in symptoms and polyp resolution in patients who received prednisolone after FESS as compared to placebo group [241]. Complete disease-free state was confirmed by nasal endoscopy in 100% patients who received oral steroids for 12 weeks. They recommended administration of postoperative oral steroid therapy for at least 12 weeks in AFRS patients. However, the exact dosage (0.4–1 mg/kg/day) and duration of oral therapy depend on the severity of symptoms and surgical outcome and need to be assessed in larger RCTs [232]. Ikram et al. noted the recurrence rate was reduced to 15% from 50% when surgery with medical therapy were combined [242]. Although the steroids have shown significant benefit in AFRS patients, their prolonged use is associated with adverse effects. On the contrary, topical corticosteroids possess a better safety profile and have shown benefit in the form of decreased polyp size and recurrence when added to local saline irrigation [189]. Rudmik et al. strongly recommended the use of standard topical steroids in patients with CRS supported by grade A evidence (well-designed randomized controlled trials (RCTs) exist and are strongly recommended) [232,243]. The evidence-based review by Gan et al. and European position paper from 2012 concluded that level 1a evidence (well-designed randomized controlled trials (RCTs) exist and are strongly recommended) exists for use of topical steroids in patients with CRSwNP although literature of their use is scarce [232]. The Food and Drug Administration (FDA) also approved the same. However, non-FDA-approved steroids should be used cautiously and restricted to refractory cases only [232].
- Antifungal therapy: There is a lack of evidence for any recommendation of oral or topical antifungal agents for AFRS [3,232]. It may be considered as an option in post-surgical refractory patients with a category C recommendation (recommended on the basis of observation studies in the form of case control and cohort) [3,232]. They may provide benefit in terms of reduction of symptoms, steroid dependence, and tendency of recurrences such as ABPA [232]. Patro et al. recently demonstrated a significant decrease in SNOT-20 and Lund Mackay scores, reduction in polyp size, fungal burden and opacification in AFRS patients who were given preoperative itraconazole for a month [244]. Similarly, Seiberling and Wornald et al. showed good response in 83% of patients using oral itraconazole 100 mg BD for 6 months after FESS [245]. Kupferberg et al. noted improved endoscopic scoring when oral antifungals were administered to AFRS patients while decreased recurrence (around 50%) and revision surgery (around 20%) were reported by Rains and Mineck using oral itraconazole [246]. Jen et al. also supported the benefits of a topical antifungal medication [247]. However, the benefits of antifungal use still need to be assessed over the adverse effects associated with systemic therapy. In addition, large, well-designed RCTs are required for proving the same.
- Immunotherapy: It aims at combating the activated adaptive immune response in AFRS patients. In 1998, Ferguson et al. described the role of immunotherapy in AFRS in a retrospective review of seven patients; five patients received immunotherapy before surgery and showed no improvement. However, the remaining two patients who were administered immunotherapy after the surgery showed good response, thereby suggesting the role of postoperative immunotherapy [248]. Following this, many reports supported the use of immunotherapy [249,250,251]. Mabry et al. concluded that immunotherapy resulted in decreased nasal crusting, decreased requirement of oral/topical steroids after 2 months and revision surgery up to 28 months follow up [249,250,251]. Folker et al. further noted overall improvement in endoscopic mucosal staging, quality of life and decreased need of steroid after 6–8 weeks’ postoperative immunotherapy [252]. Bassichis et al. also found similar results in addition to decreased need of revision surgery [253]. However, Marple et al. in 2002 failed to show any significant benefit of immunotherapy, thereby questioning its role in management [254]. Its use in the form of subcutaneous application is devoid of any local or systemic side effects [255]. Therefore, immunotherapy may serve as adjunct therapy in refractory cases without any unusual adverse event or formation of immune complexes although the data is limited to case reports and retrospective studies [232]. With the level of evidence C (only observation studies in the form of case control and cohort available), its recommendation is still challenging [232].
- Leukotriene modulators: There is no controlled study available regarding use of these agents in AFRS. There is only one case report of successful postoperative management of AFRS with montelukast 10 mg daily along with topical corticosteroids [256]. However, these agents have shown mixed results in other types of CRSwNP with either improved symptoms and CT scores or no benefit in comparison to steroids [233].
- Others: Anti IL-5 antibody (mepolizumab) may help to reduce polyp size and sinus opacification, as observed in a randomized controlled trial (RCT) [257]. However, the role of reslizumab in nasal polyposis is still being explored [258]. Gan et al. administered omalizumab, which binds selectively to IgE causing decrease in its levels of both serum and tissue in seven refractory cases of AFRS [259]. They observed 31% improvement in Sino-Nasal Outcome Test-22 (SNOT-22) score (52.14 decreased to 35.86) and 61% improvement in Phillpott-Javer endoscopic score (36 to 14). Omalizumab therapy also reduced the dependence of AFRS patients on corticosteroid and antifungal treatments [232]. There is also a case report of successful outcome of AFRS refractory to FESS and systemic corticosteroids with omalizumab [260]. In addition, antibacterial therapy like mupirocin has been proposed for local use to reduce both planktonic and biofilm forms of *S. aureus* which act as disease-modifying agents [261]. However, lack of evidence creates a dilemma about its use. Other possible therapeutic targets include TSLP inhibitors and P glycoprotein inhibitors [178,262].

Overall, both oral and topical steroids postoperatively are believed to be the choice of therapy. Antifungals and immunotherapy may serve as adjuncts in recalcitrant cases.

## 10. Conclusions

The review provides an update on allergic fungal rhinosinusitis (AFRS), a unique entity subject to a great deal of controversy in classification, pathogenesis, diagnostic criteria, and management protocols. The diagnosis of AFRS combines clinical, radiological, microbiological, and pathological observations. The disease appears to be a complex interplay of IgE-mediated systemic/local hypersensitivity to fungal antigens, host defense mechanisms (innate and adaptive including both T cell and B cell-mediated immune responses), and possibly superantigens. The differential gene expression in AFRS and eosinophilic mucin rhinosinusitis (EMRS) needs to be elucidated as mentioned earlier [3]. Some of these genes are shown to be associated with autoimmunity and malignancy and their role needs to be further explored. The role of fungi in initiating or maintaining the disease process remains controversial. The management of AFRS is largely surgical along with an important role for oral corticosteroids and an emerging role for immunotherapy and antifungals in recalcitrant cases. The role of leukotriene antagonists needs more evidence. Molecular studies are needed to unravel the mechanisms of infiltration, activation, and maintenance of immune response for targeted therapy.
